# Podocyte-Specific Deletion of Murine CXADR Does Not Impair Podocyte Development, Function or Stress Response

**DOI:** 10.1371/journal.pone.0129424

**Published:** 2015-06-15

**Authors:** Christoph Schell, Oliver Kretz, Andreas Bregenzer, Manuel Rogg, Martin Helmstädter, Ulrike Lisewski, Michael Gotthardt, Pierre-Louis Tharaux, Tobias B. Huber, Florian Grahammer

**Affiliations:** 1 Renal Division, University Medical Center Freiburg, Freiburg, Germany; 2 Spemann Graduate School of Biology and Medicine (SGBM), Albert-Ludwigs University Freiburg, Freiburg, Germany; 3 Faculty of Biology, Albert-Ludwigs University Freiburg, Freiburg, Germany; 4 Department of Neuroanatomy, Albert-Ludwigs University Freiburg, Freiburg, Germany; 5 Max-Delbrueck Center for Molecular Medicine, Berlin, Germany; 6 Paris Centre de Recherche Cardiovasculaire, INSERM, Paris, France; 7 BIOSS Center for Biological Signaling Studies, Albert-Ludwigs-University Freiburg, Freiburg, Germany; Institut National de la Santé et de la Recherche Médicale, FRANCE

## Abstract

The coxsackie- and adenovirus receptor (CXADR) is a member of the immunoglobulin protein superfamily, present in various epithelial cells including glomerular epithelial cells. Beside its known function as a virus receptor, it also constitutes an integral part of cell-junctions. Previous studies in the zebrafish pronephros postulated a potential role of CXADR for the terminal differentiation of glomerular podocytes and correct patterning of the elaborated foot process architecture. However, due to early embryonic lethality of constitutive *Cxadr* knockout mice, mammalian data on kidney epithelial cells have been lacking. Interestingly, *Cxadr* is robustly expressed during podocyte development and in adulthood in response to glomerular injury. We therefore used a conditional transgenic approach to elucidate the function of *Cxadr* for podocyte development and stress response. Surprisingly, we could not discern a developmental phenotype in podocyte specific *Cxadr* knock-out mice. In addition, despite a significant up regulation of CXADR during toxic, genetic and immunologic podocyte injury, we could not detect any impact of *Cxadr* on these injury models. Thus these data indicate that in contrast to lower vertebrate models, mammalian podocytes have acquired molecular programs to compensate for the loss of *Cxadr*.

## Introduction

The renal filtration barrier is composed of at least four layers, the endothelial glycocalyx, the endothelial fenestrae, the glomerular basement membrane and the slit diaphragm (SD) in between neighbouring podocyte foot processes [1]. This last part of the filter is composed of a variety of different types of intercellular junctions, forming a uniquely broad, permeable and still highly selective barrier. Beside slit-diaphragm specific components such as NEPHRIN, NEPH1 and PODOCIN, adherens junctional proteins i.e. CADHERIN or CATENINs as well as tight junctional components i.e. JAM-A, OCCLUDIN, CINGULIN and ZO-1 have been identified and localized to this junction [2–5]. Interestingly several members across different junctional classes belong to the Immunoglobulin Superfamily (IgSF) of molecules: NEPHRIN, NEPH1 and JAM-A [6].

Work on normal and nephrotic rat glomeruli identified the coxsackie- and adenovirus receptor (CXADR) as another IgSF member of the podocyte SD, which was upregulated in puromycin aminonucleoside (PAN) treated rats [7, 8]. In addition, it was demonstrated that CXADR physically interacts with SD proteins such as PODOCIN [7]. Furthermore a morpholino based CXADR knock-down approach in zebrafish suggested a role for CXADR in the terminal differentiation of glomerular podocytes [9].

CXADR was identified in 1997 as the receptor mediating binding and uptake of Coxsackie type B viruses and adenovirus type 2 and 5 [10, 11]. Subsequently it was shown that CXADR is an integral component of tight-junctions and interacts with ZO-1, MAGI-1 and MUPP1 [12–14]. Constitutive *Cxadr* knock-out mice exhibited an early embryonic lethality between E11,5 and E13,5 due to heart abnormalities [15]. Further evaluation using an inducible conditional mouse genetic approach, revealed that loss of CXADR results in impaired electrical conduction between the cardiac atrium and ventricle, which was underlined by a functional interaction of CXADR with connexins [16].

To elucidate the precise role of CXADR for mammalian podocyte development, maintenance and stress response *in vivo* we analyzed podocyte specific conditional *Cxadr* knockout mice.

## Materials and Methods

### Animals

All animal experiments were conducted according to the National Institutes of Health *Guide for the Care and Use of Laboratory Animals*, as well as the German law for the welfare of animals and were approved by local authorities [G-09/23 Regierungspräsidium Freiburg]. Mice were generated as previously described [16] and crossed using the *Tg(hNPHS2-cre)295Lbh* line [17]. Mice were housed in a SPF facility with free access to chow and water and a 12h day/night cycle. Breeding and genotyping was done according to standard procedures. To assess genetic influence on CXADR expression the well described *Cd2ap-/-* on a C57Bl6 background was used at the age of 4 weeks [18].

### Developmental assessment

Individual age and sex matched animals from parallel litters on a C57Bl6/NCrl background were used. Control animals (*Cxadr fl/fl*) were compared with podocyte specific (Cxadr fl/fl*hNphs2Cre) mice. Collection of spot urine samples in the respective home cages was performed between 7 to 9 am at defined time points as indicated. Urinary albumin and creatinine were measured using a fluorimetric albumin test kit (Progen, PR2005, Heidelberg, Germany) and enzymatic colorimetric creatinine kit (LT-SYS, Lehmann, Berlin, Germany) following the manufacturer´s instructions. Evaluation of proteinuria (expressed as the albumin to creatinine ratio) was performed as previously described [19–21]. Primary outcome was development of proteinuria.

### Glomerular stress models

For some of the following stress experiments C57Bl6/NCrl mice were backcrossed to ICR mice (Taconic, New York, USA) for at least 6 generations as indicated in the result section. All other experiments were performed on a C57Bl6/NCrl background. We used the following well established stress-models: A.) adriamycin, and B.) nephrotoxic serum (NTS). In each of these, control animals (*Cxadr fl/fl*) were compared with podocyte specific (Cxadr fl/fl*hNphs2Cre) mice. Animals were allocated to the two groups based on age and sex. A.) Adriamycin (Pharmacy, University Hospital Freiburg, Germany) was administered at a dose of 15 μg/g BW in 0,9% saline i.v. under isoflurane anesthesia after collection of control urine. Urine was again collected at 1, 2, 3, 4 and 5 weeks after injection. Mice were killed by cervical dislocation and kidneys harvested at 5 weeks after injection. B.) NTS of sheep origin was generously supplied by Pierre-Louis Tharaux (INSERM PARCC, Paris, France) and generated as previously described [22]. Briefly, mice were injected with 2μl/g BW NTS on day 0, 6μl/g BW on day 1 and 5μl/g BW on day 2. each time under isoflurane anesthesia. Urine was collected before the respective injection on day 0 and 2 and additionally on day 3 and 4. Due to the severity of the phenotype and in accordance with our animal proposal mice were killed by cervical dislocation on day 4 and kidneys were harvested.

### Isolation of glomeruli and podocytes

We used the magnetic bead method described by Takemoto *et al*. 2006 with appropriate modifications [23]. Briefly, kidneys were dissected together with the abdominal aorta and transferred into dishes filled with 37°C pre-warmed Hank’s buffered salt solution (HBSS). Each kidney was perfused slowly through the renal artery with 4 ml 37°C warm bead solution and 1 ml bead solution plus enzymatic digestion buffer [containing: collagenase 300 U/ml (Collagenase Type II, Worthington, Lakewood, New Jersey, USA), 1 mg/ml pronase E (P6911, Sigma, Schnelldorf, Germany) DNase I 50 U/ml (A3778, Applichem, Darmstadt, Germany)]. Kidneys were minced into 1 mm³ pieces using a scalpel. After addition of 3 ml digestion buffer they were incubated at 37°C for 15 min on a rotator (100rpm). The solution was pipetted up and down with a cut 1000μl pipette tip every 5 min. After incubation all steps were performed at 4°C or on ice. The digested kidneys were gently pressed twice through a 100 μm cell-strainer and the flow through was washed extensively with HBSS. After spinning down, the supernatant was discarded and the pellet resuspended in 2 ml HBSS. These tubes were inserted into a magnetic particle concentrator and the separated glomeruli were washed twice. Podocytes were isolated as previously described [24].

### Morphological analysis

Kidneys were perfusion fixed in 4% phosphate buffered paraformaldehyde (Sigma, Schnelldorf, Germany), embedded in paraffin and further processed for PAS staining. For ultrastructural transmission electron microscopy (TEM) analysis kidneys were fixed in 4% phosphate buffered paraformaldehyde plus 1% glutaraldehyde (Serva, Heidelberg Germany). Samples were postfixed in 1% osmium tetroxide in the same buffer for 1 hour and stained *en bloc* in 1% uranyl acetate in 70% ethanol for 1 hour, dehydrated in ethanol, and embedded in Durcopan (Plano, Wetzlar, Germany). Thin sections were stained with lead citrate and examined in a Zeiss Leo-906 transmission electron microscope. For scanning electron microscopy samples were fixed with 4% glutaraldehyde for 4 days and were then subsequently dehydrated (EtOH 50, 70, 80, 90 and 100%; 1:1 EtOH and Hexamethyldisilazan (HMDS) (Sigma, Schnelldorf, Germany) for 1 hour and 30 minutes 100% HMDS, afterwards solvent was allowed to evaporate) and coated with Gold (Zeiss Semco Nanolab7, Polaron Cool Sputter Coater E 5100, Balzer Cpd 020) [25]. Image acquisition was performed using a Leo 1450 VP scanning electron microscope.

### Western Blot and Immunofluorescence

Kidneys or isolated glomeruli were glass-glass-homogenized in lysis buffer (containing 20 mM CHAPS and 1% Triton X-100). After centrifugation (15,000x g, 15 min, 4°C) protein concentration was determined by DC Protein-Assay (Bio-Rad, Hercules, California, USA). Equal amounts of protein were separated on SDS page. For immunofluorescence kidneys were frozen in OCT compound and sectioned at 4–5 μm (Leica Kryostat, Leica, Wetzlar, Germany). The sections were fixed with 4% paraformaldehyde, blocked in PBS containing 5% BSA + 5% Normal Donkey Serum (Jackson ImmunoResearch, Suffolk, Great Britain) and incubated for 45 min with primary antibodies as indicated. For co-stainings with CXADR initial fixation was performed using methanol at -20° for 10 minutes. After several PBS rinses, fluorophore-conjugated secondary antibodies (Life Technologies, Darmstadt, Germany) were applied for 30 minutes. Image acquisition was done either using a confocal imaging set up (Zeiss LSM 510 upright microscope, Zeiss, Germany), equipped with a Plan-Apochromat 63x/1.4 Oil M27 objective or a epifluorescence widefield imaging setup (Zeiss Axioplan 2 upright + Axiocam MRc5 digital camera)). Image recording was performed with the appropriate software (Zen black Software or Axiovision LE46—Zeiss). The following antibodies were used: anti-CXADR (HPA030411, Atlas Antibodies, Stockholm, Sweden), anti-CXADR (H300, sc15405, Santa Cruz Biotechnology, Heidelberg, Germany) anti-NIDOGEN (Clone ELM1, Millipore, Darmstadt, Germany), anti-NEPHRIN (GP-N2, Progen, Heidelberg, Germany), anti-ZO-1 (Clone ZO1-1A12, Life Technologies, Karlsruhe, Germany), anti-β-Actin (Clone AC-15, Sigma, Schnelldorf, Germany), anit-CD2AP (generous gift by Andrey Shaw, Washington University, St. Louis, USA), FITC-LTG and Texas-Red- DBA (Vectorlabs, California, USA), Hoe33342 and respective Alexa-Fluor 2^nd^ antibodies (all Life Technologies, Karlsruhe, Germany).

### In-Situ Hybridization

A mouse kidney cDNA library served to clone a fragment of the coding sequence of mouse *Cxadr*. The following primers were used: *ISHcxadrMluF CGCGGGACGCGTACCAGGGACCACTGGACATT* and *ISHcxadrNotB1*: *CGCGGGGCGGCCGCGCGCACGTTCAAAGTCTTCA*, yielding a 843 bp PCR product. PCR fragments were inserted into *pBluescript SK*+ vector (Invitrogen, Carlsbad, CA, USA) using Not I and Mlu I restriction sites. *pBluescript SK*+ vector was linearized and digoxigenin-(DIG)-labeled antisense riboprobes were generated using T7-RNA-polymerase (Ambion, Karlsruhe, Germany). For paraffin ISH sections, slides were progressively rehydrated and permeabilized with proteinase K for 3 min. After prehybridization (60 min), hybridization with DIG-UTP probes took place overnight in standard saline citrate (SSC; pH 4.5; containing 50% formamide) at 65–70°C. Specimens were then incubated with alkaline phosphatase-conjugated anti-DIG Fab fragments (Roche, Mannheim, Germany) at a dilution of 1:4,000 overnight at 4°C. Alkaline phosphatase was detected using chromogenic conversion of NBT/BCIP (Roche, Mannheim, Germany). To avoid drying up of the slides during hybridization, we placed them in a humidity chamber containing 5×SSC and 40% formamide. Slides were then progressively dehydrated, washed in xylol, and mounted.

### Statistics

Data are expressed as mean ± SEM. Statistical comparisons were performed using the GraphPad Prism Software Package (Ver.6, GraphPad Software, La Jolla, California, USA) with two-tailed Student’s t-test or ANOVA including respective corrections where indicated. Differences with p values below 0.05 were considered significant.

## Results

### *Cxadr* is highly expressed during kidney development.

**Fig 1:**
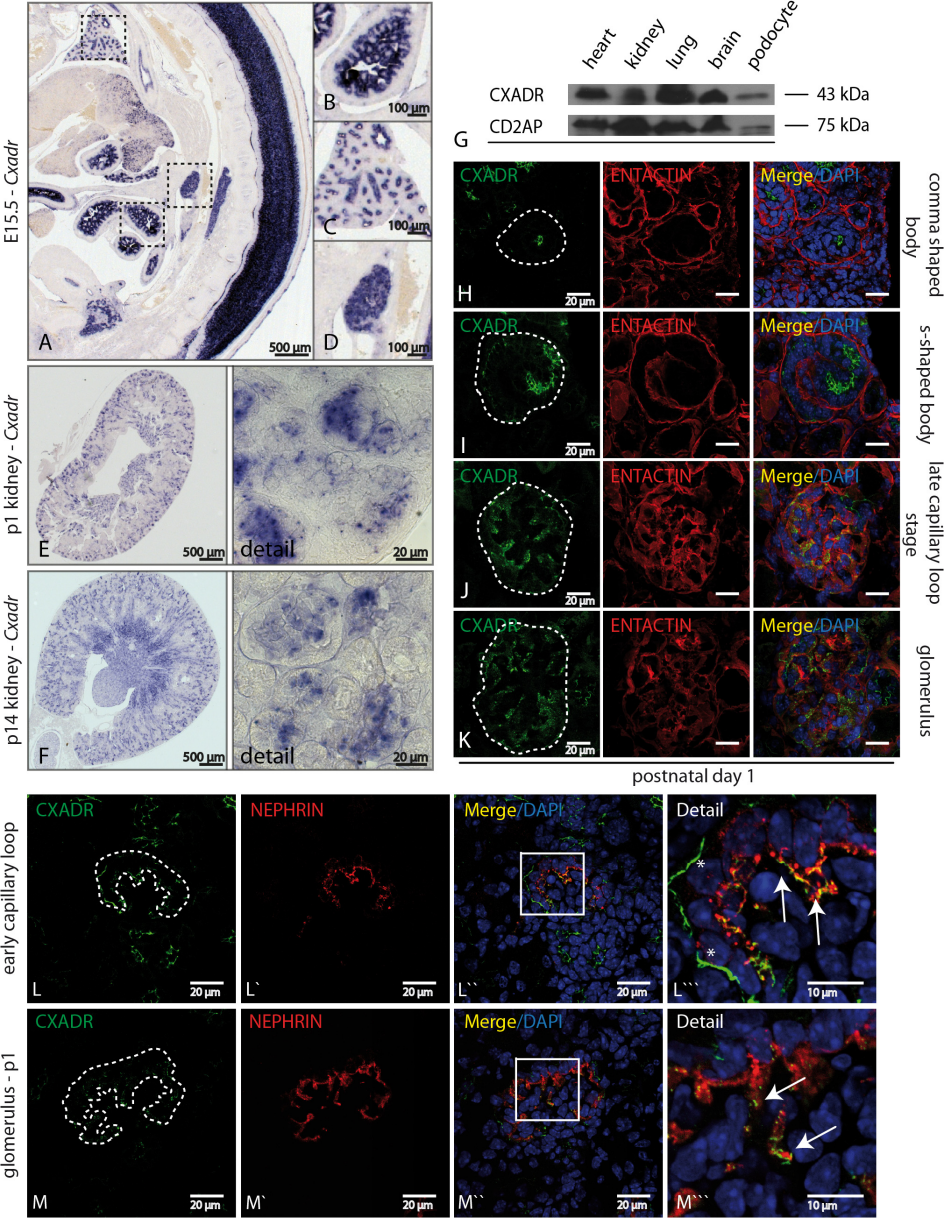
*Cxadr* is highly expressed during kidney development. **(A-D)** In-Situ-Hybridization showed intense expression of *Cxadr* in E15.5 embryos, especially in the nervous system, gut, lung and kidney. **(E&F)** After birth at P1 and P14 *Cxadr* could be detected in glomerular cells as well as in the nephron (*thick ascending limb of Henle*). **(G)** Western blot confirmed CXADR expression in heart, kidney, lung, brain and more specifically in isolated murine podocytes. **(H-K)** Developmental assessment of *Cxadr* expression during kidney development started in the S-shaped body phase where podocytes and parietal epithelial cells formed a continuous stretch of cells. **(L&M)** In both, podocytes (arrows) and parietal epithelial cells (asterisk) CXADR could be robustly detected in the early capillary loop stage, while mature glomeruli only possessed reduced amounts of CXADR in podocytes.

*Cxadr* expression was analyzed using in-situ hybridization. Beside a predominant CNS signal, *Cxadr* was strongly expressed in several epithelial tissues including gut, lung and kidney (Fig 1A, 1B, 1C and 1D). Within the kidney *Cxadr* was localized to glomerular podocytes and epithelial cells of several tubular segments including the proximal tubule and the thick ascending limb of Henle (Fig 1E and 1F +S1 Fig). Western blot analysis of 6 week old animals confirmed protein expression in heart, kidney, lung, brain and isolated murine primary podocytes (Fig 1G). P1 kidneys were used to analyze the *Cxadr* expression during glomerular maturation (Fig 1H, 1I, 1J and 1K): In the S-shaped body stage CXADR was first expressed in epithelial cells committed to become parietal epithelial cells and podocytes, but not in cells destined to become proximal tubular cells (Fig 1H). Podocytes and parietal epithelial cells continued to express CXADR while mesangial and endothelial cells never showed a detectable signal (Fig 1J and 1K + S1 Fig). From the early capillary loop stage to mature glomerula CXADR co-localized with the SD protein NEPHRIN (Fig 1L and 1M). In addition, a strong linear signal of CXADR along Bowman’s capsule, representing CXADR expression in parietal epithelial cells, was observed (Fig 1L”`, white asterisks). Interestingly, the expression of CXADR in podocytes vanished by 6 weeks of age, while parietal epithelial cell expression remained constantly high (S1C–S1E Fig).

### Podocyte specific knock-out of *Cxadr* does not impair glomerular development.

**Fig 2:**
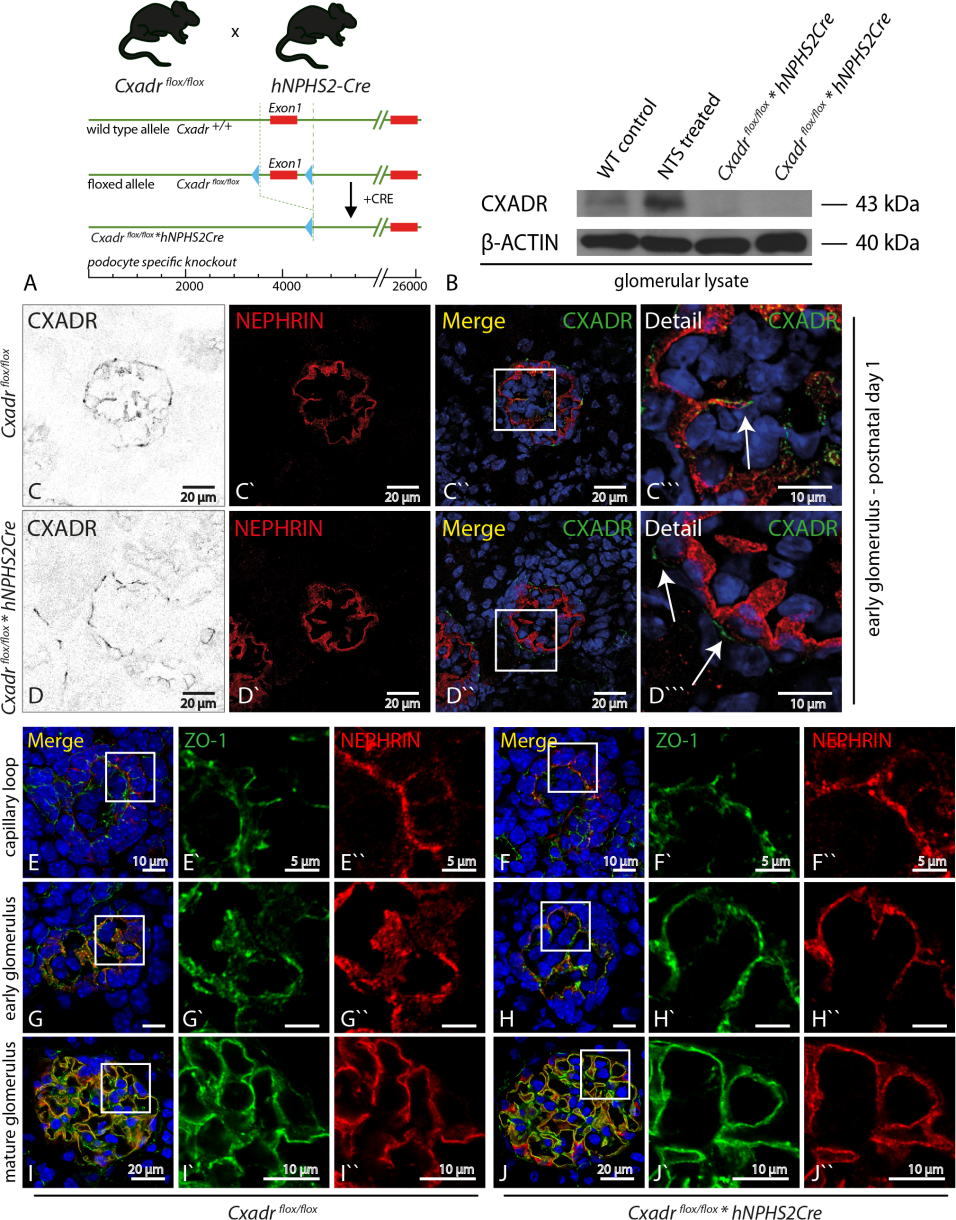
Podocyte specific knock-out of *Cxadr* does not impair glomerular development. **(A)** Targeting strategy leading to podocyte specific deletion of Exon 1. **(B)** In decapsulated wild-type glomeruli CXADR protein could be detected by western blot. NTS stimulates CXADR expression. In podocyte specific *Cxadr* deficient animals no protein could be detected, indicating that all detected glomerular CXADR protein is of podocyte origin. **(C&D)** Immunofluorescence analysis showed co-localization of CXADR and NEPHRIN in wild-type animals, which was lost in podocyte specific knock-out animals. CXADR expression in parietal epithelial cells (arrows in D”‘) was unaffected by our targeting approach. **(E-J)** Analysis of ZO-1 and NEPHRIN expression at three decisive time points during glomerular development in control and *Cxadr* podocyte specific knock-out animals reveals no difference with regard to expression abundance and distribution of both a slit-diaphragm and a tight junctional protein.

**Fig 3:**
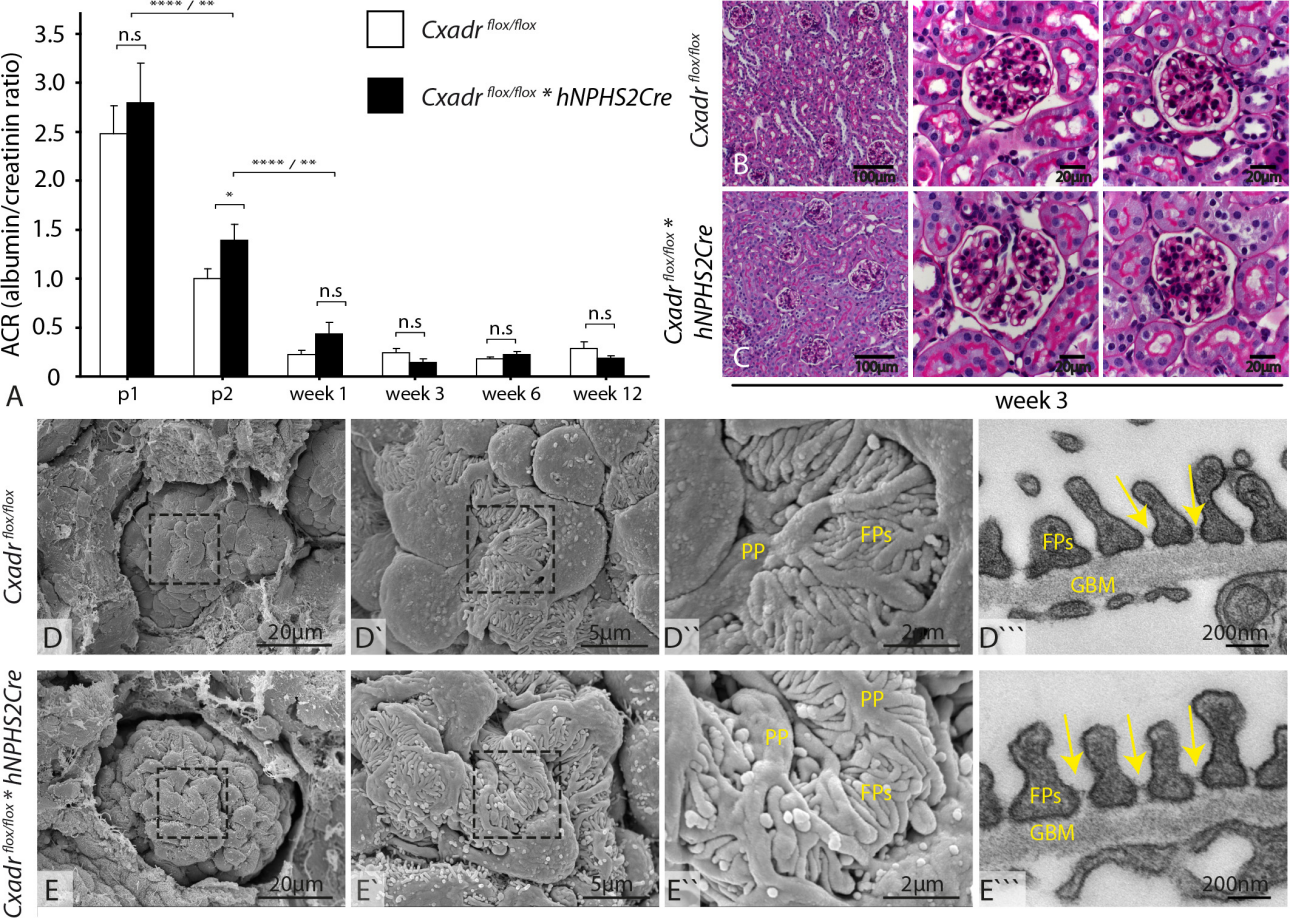
Glomerular function, histology and ultrastructure are maintained in podocyte specific *Cxadr* knock-out animals. **(A)** Functional assessment only showed a slightly elevated albuminuria in *Cxadr* deficient animals at P2 which was lost during further maturation of the kidney. **(B&C)** Histology of glomeruli was normal in podocyte deficient *Cxadr* animals as was **(D&E)** ultrastructural assessment by SEM **(D–D”**, **E–E”)** and TEM **(D”‘**, **E”‘)**.

We generated podocyte specific *Cxadr* deficient animals, using the established *hNPHS2Cre* system (Fig 2A). Westernblot of glomerular lysates and immunofluorescence staining confirmed CXADR expression in wild-type animals, which was up-regulated upon NTS treatment and absent in podocyte specific *Cxadr* deficient mice (Fig 2B, 2C and 2D). The specificity of our conditional approach was demonstrated by the maintained robust parietal epithelial cell expression of CXADR in both genotypes. (Fig 2D”`—white arrows). Using immunofluorescence we evaluated the abundance and distribution of the slit diaphragm proteins NEPHRIN and PODOCIN as well as of the tight junctional protein ZO-1. At all three developmental stages examined–capillary loop stage, early and mature glomerulus glomerulus—we neither found any decisive difference in staining intensity nor any difference in protein distribution within podocytes of control and podocyte–specific *Cxadr* knock-out animals (Fig 2E–2J, S2A–S2F Fig). We therefore conclude that absence of CXADR does not lead to any major detectable changes in NEPHRIN, PODOCIN and ZO-1, at least with our methods used. Albumin excretion between genotypes was only slightly significantly different at P2 but not on all other five tested time points (Fig 3A, n = 5–10 per time point and group). Similarly, light microscopy at week 3, as well as SEM and TEM did not show any obvious histological or ultrastructural abnormalities (Fig 2B–2E).

### Adriamycin enhances podocyte specific CXADR expression, but lack of CXADR does not influence the course of Adriamycin induced FSGS.

**Fig 4:**
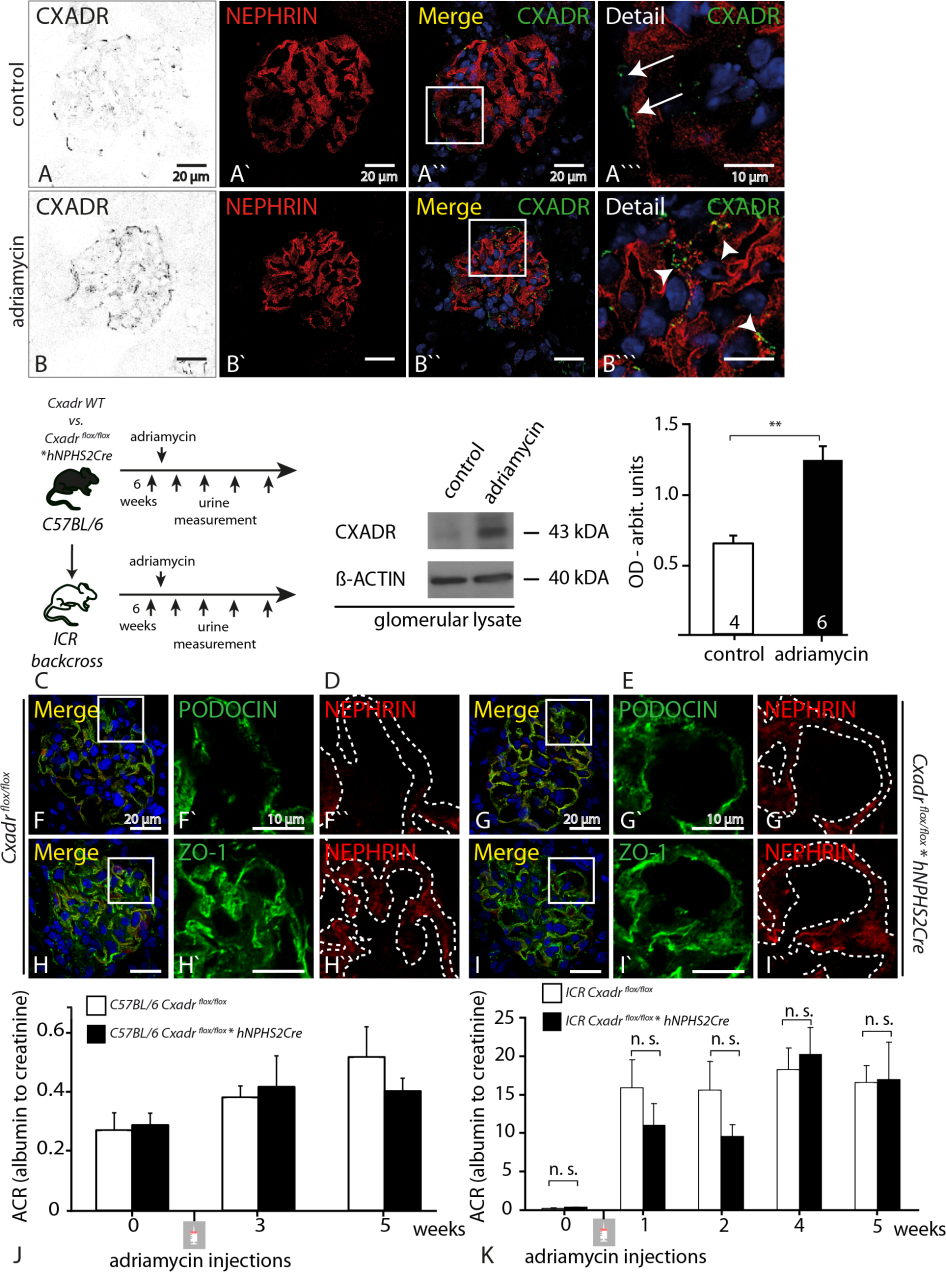
Adriamycin enhances podocyte specific CXADR expression, but lack of CXADR does not influence the course of Adriamycin induced FSGS. **(A)** In adult wild-type animals CXADR was restricted to parietal epithelial cells (white arrows). **(B)** Administration of Adriamycin led to an increased glomerular CXADR expression which could be localized to glomerular podocytes using co-labeling experiments with NEPHRIN (white arrow heads). **(C)** Adriamycin injection scheme and subsequent urine measurements. **(D&E)** Western blots of decapsulated glomerular lysates were used to quantify CXADR expression in podocytes which indeed was upregulated two-fold. We next assessed abundance and distribution of the slit diaphragm molecules PODOCIN and NEPHRIN after adriamycin injection, and could not discern any differences between the two genotypes **(F-F”, G-G”** dashed lines-podocyte compartment**)**. In contrast to NEPHRIN the pattern of ZO-1 expression was largely maintained in both genotypes. **(H-H”, I-I”** dashed lines—podocyte compartment**).** We next assessed functional consequences on two different mouse genetic background strains. **(J)** Wild-type and knock-out animals on a C57Bl6/NCrl were analysed to check for increased susceptibility of knock-out animals while **(K)** wild-type and knock-out animals on an ICR background were used to test for a protective effect of podocyte specific CXADR deficiency. On both mouse genetic backgrounds we could not discern a difference between wild-type and knock-out mice over a follow-up period of 5 weeks.

Next we exposed control and podocyte deficient animals to adriamycin. This anthracycline antibiotic is known to cause proteinuria and a FSGS like phenotype when administered to mice on a sensitive genetic background (e.g. ICR, balb/c), while it does not cause an overt podocyte phenotype in resistant mouse strains (i.e. C57Bl6/NCrl) [26]. Hence, depending on the genetic background the adriamycin stress model allows to test for either sensitization or protection in the context of the respective gene knockout. Using immunofluorescence and western blot we were able to demonstrate that adriamycin upregulates CXADR in control podocytes (Fig 4A, 4B, 4D and 4E). Inversely to CXADR expression, the expression level of the slit diaphragm molecules NEPHRIN and PODOCIN was greatly reduced in both genotypes (Fig 4F and 4G–staining was performed on ICR backcrossed animals of both respective genotypes). This was in contrast to ZO-1 which showed virtually constant expression levels and equal distribution between health and disease in all mice (Fig 4H and 4I). However, despite profound up-regulation of CXADR on both genetic backgrounds we could not identify any clinical functional difference between genotypes over the course of five weeks (Fig 4C, 4J and 4K; C57Bl6/NCrl background: control group n = 3, Cxadr fl/fl*hNphs2Cre n = 4; ICR background: control group n = 6, Cxadr fl/fl*hNphs2Cre n = 7).

### Nephrotoxic serum (NTS) enhances podocyte specific CXADR expression, but lack of CXADR does not influence the course of NTS induced disease.

**Fig 5:**
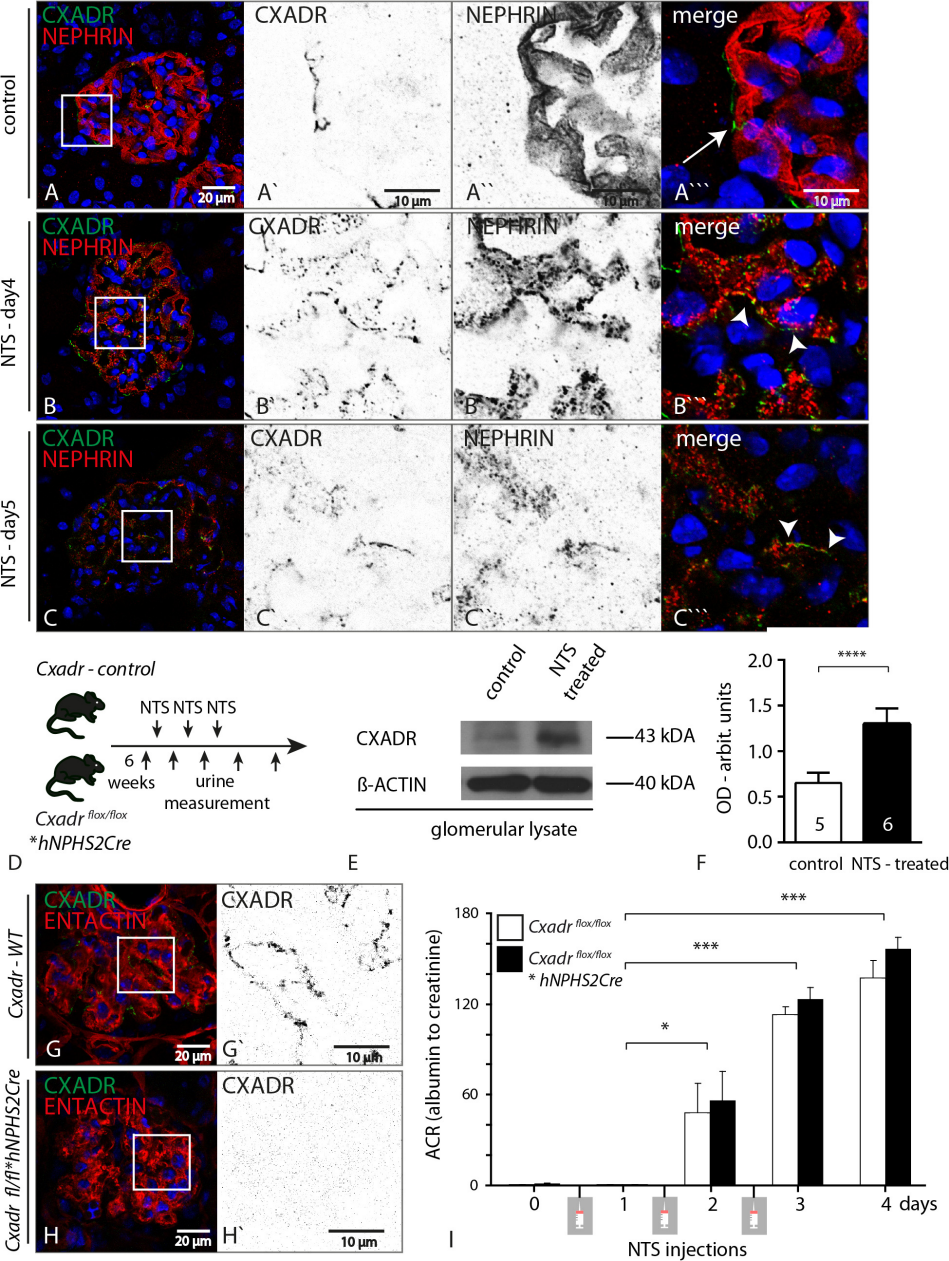
Nephrotoxic serum (NTS) enhances podocyte specific CXADR expression, but lack of CXADR does not influence the course of NTS induced disease. **(A-C)** On both d4 and d5 after NTS injection CXADR was increased in glomerular podocytes as shown in co-labeling experiments with NEPHRIN (control: white arrows—parietal epithelial cells, NTS: arrow heads—podocytes). As can be easily depicted from the NEPHRIN specific panel **(A”, B”, C”)** NEPHRIN abundance is greatly reduced during the course of the disease, underlining the severity of the chosen stress model. **(D)** Schematic of the injection scheme and follow-up using urine collections. **(E&F)** Western blot using decapsulated glomerular lysates was used to quantify CXADR expression which was increased two fold 5 days after NTS injection. **(G&H)** As shown with immunofluorescence stainings CXADR induction was absent in podocyte specific *Cxadr* knock-out animals. **(I)** Proteinuria developed similarly in wild-type and knock-out animals showing no functional differences.

Subsequently we examined whether CXADR up-regulation is limited to toxic nephrotic states or whether this also applies to genetic or immunological conditions. Indeed, using an established genetic model of nephrotic syndrome (*Cd2ap* knockout animals) and sheep derived nephrotoxic serum (NTS), we could demonstrate that in both conditions tested, CXADR was strikingly up-regulated (Fig 5A–5C, 5E and 5F + S3A and S3B Fig). We therefore treated wild-type and *Cxadr* podocyte deficient animals with NTS, but again despite marked proteinuria within 4 days after administration of NTS, we could not detect any difference between respective genotypes (Fig 4D, 4G, 4H and 4I; control group n = 6, Cxadr fl/fl*hNphs2Cre n = 6).

## Discussion

Podocyte function and maintenance is regulated by precisely orchestrated cell-cell contacts. The discovery of the molecular composition of the SD has profoundly changed our understanding of glomerular filtration and function. However, the dynamic changes, molecular make up and intermolecular interactions at the SD remain incompletely understood. Here we identify the IgSF member CXADR to be specifically regulated and expressed in developing and injured mammalian podocytes. CXADR shows an early and specific expression in precursors of podocytes and parietal epithelial cells, starting in the S-shape body phase during glomerular development. Yet, while podocyte specific expression vanishes over the first six weeks of life, expression in parietal epithelial cells stays prominent through-out life. Given the potential role of parietal epithelial cells for podocyte regeneration and repair, this might indicate that *Cxadr* is a marker of immature podocytes which is lost during podocyte differentiation [27]. This loss of CXADR is actually paralleled by the formation of the slit-diaphragm out of a tight-junction based precursor [1]. Developmental reduction of *Cxadr* expression levels seems to be a common theme and has been described for brain and heart before [28, 29].

Interestingly, and in contrast to previously published results in the zebrafish pronephros [9], conditional podocyte specific knock-out in mice does not lead to a developmental phenotype. Obviously, it is known from previous studies that the *hNPHS2* promotor is only active from approximately embryonic day E 14.0 onwards when glomerular development proceeds from the S-shaped body towards the early capillary loop stage [30]. As mentioned, CXADR is already highly expressed in the S shaped stage and hence early essential roles might be missed by a relatively late recombination event. Yet, *hNPHS2-Cre* mediated knock-out seems efficient with regard to CXADR protein reduction, as we could not detect any protein anymore in late capillary loop stages of glomerular development in respective knock-out animals.

As shown with different types of toxic, genetic and immunological glomerular injury models, *Cxadr* expression is robustly upregulated during podocyte injury. It could therefore be used as a podocyte stress marker, similarly to down regulation of SESTRIN in parietal epithelial cells [31, 32]. Interestingly, upregulation of CXADR during disease has been previously described in the heart under various conditions i.e. autoimmune inflammation, infarction and dilated cardiomyopathy [29, 33, 34]. A potential functional role of CXADR up regulation in response to glomerular injury is unfortunately not being revealed by our data. In fact, our analysis indicates that presence or absence of podocyte CXADR does not change the course of injury or repair, at least within the models and time lines evaluated in this study. In addition expression and distribution of other decisive slit diaphragm and tight junctional molecules was not changed. This in our opinion at least allows two conclusions: 1. There seems to be a redundancy with regard to Super Ig cell adhesion molecules possessing only 2 extracellular IgG domains and other members expressed in podocytes could potentially take over CXADR’s function [24, 35]. 2. ZO-1 based tight junctions forming during podocyte injury do not rely on additional CXADR expression and form stable cell-cell contacts themselves.

As CXADR expression within the glomerulum is much more prominent in parietal epithelial cells, future studies might be warranted to address the role of CXADR in these specialized epithelial cells of Bowman’s Capsule. This not only applies to their unchallenged physiologic state but also to any changes which occur in glomerular diseases such as rapid progressive glomerulonephritis, where proliferation and crescent formation of parietal epithelial cells are distinguished hallmarks.

## Supporting Information

S1 ARRIVE Checklist Supplemental Information ARRIVE. A completed ARRIVE checklist concerning all animal experiments in this manuscript is given.
(PDF)

S1 Fig Non-podocyte renal expression of CXADR. **(A&B)** CXADR was expressed in proximal as well as distal parts of the renal tubular system, as demonstrated by co-labeling with either LTG or DBA-lectins. Here CXADR localizes clearly to cell-cell contacts of renal tubular epithelial cells. **(C-E)** Immunofluorescence at different developmental time points demonstrated a reduction of podocyte CXADR expression, whereas a strong signal was still present in parietal epithelial cells of adult animals.
(TIF)

S2 Fig Developmental expression of the bona fide slit diaphragm proteins NEPHRIN and PODOCIN in control and podocyte specific *Cxadr*-/- mice. Assessment of NEPHRIN and PODOCIN expression at different developmental stages–capillary loop, early glomerulus, mature glomerulus–in control (A-A”, C-C”, E-E”) and podocyte specific *Cxadr-/-* animals (B-B”, D-D”, F-F”) was performed using immunofluorescence. Loss of CXADR does not lead to changes in abundance and distribution of neither NEPHRIN nor PODOCIN.
(TIF)

S3 Fig CXADR is also upregulated in podocytes in the *Cd2ap-/-* model of genetic glomerular disease. **(A&B)** CD2AP knockout animals exhibited a clear upregulation of CXADR expression in podocytes as demonstrated by immunofluorescence staining of CXADR and respective co-labeling with NEPHRIN.
(TIF)
