# Supplementary figures and images for: Podocyte-Specific Deletion of Murine CXADR Does Not Impair Podocyte Development, Function or Stress Response
Source: PLoS One. 2015 Jun 15;10(6):e0129424. doi: 10.1371/journal.pone.0129424 (PMC4468136; doi:10.1371/journal.pone.0129424)

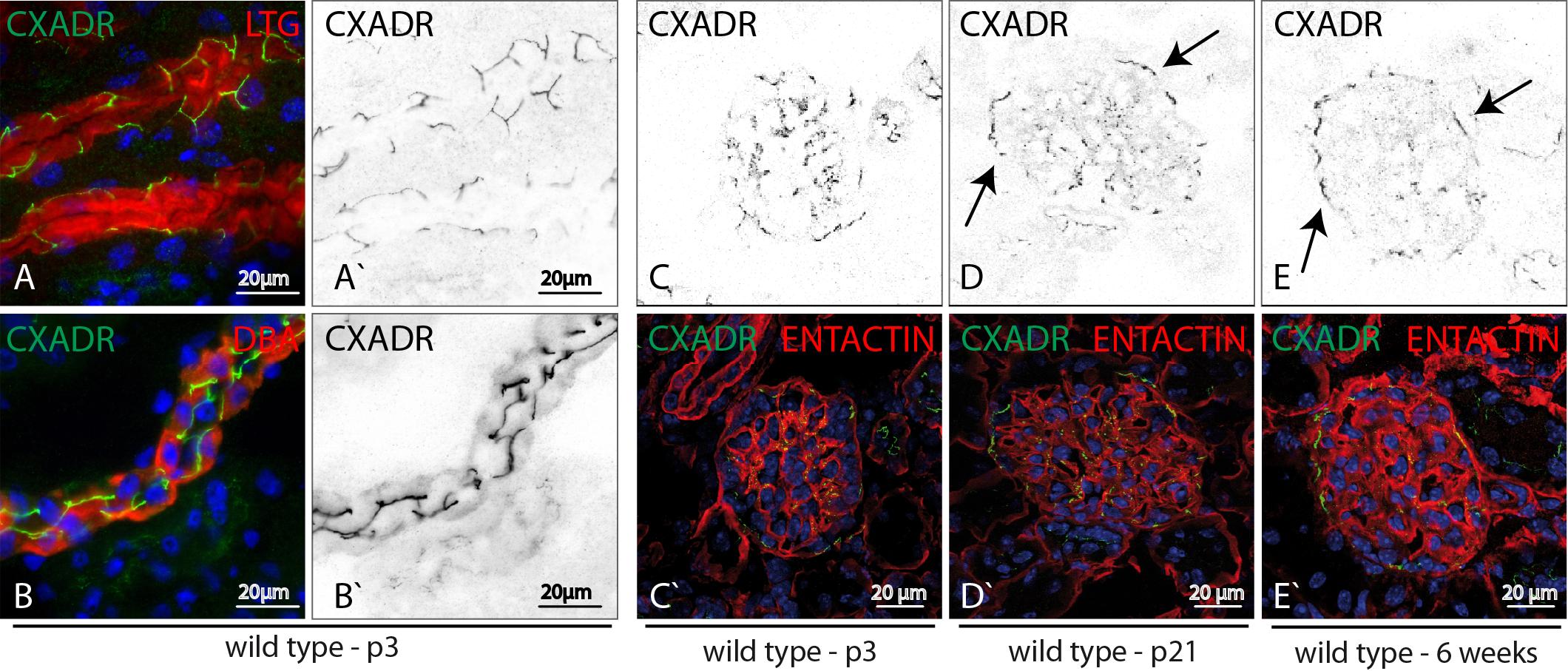

Supplement: S1 Fig — (A&B) CXADR was expressed in proximal as well as distal parts of the renal tubular system, as demonstrated by co-labeling with either LTG or DBA-lectins. Here CXADR localizes clearly to cell-cell contacts of renal tubular epithelial cells. (C-E) Immunofluorescence at different developmental time points demonstrated a reduction of podocyte CXADR expression, whereas a strong signal was still present in parietal epithelial cells of adult animals. (TIF) [file pone.0129424.s002.tif]

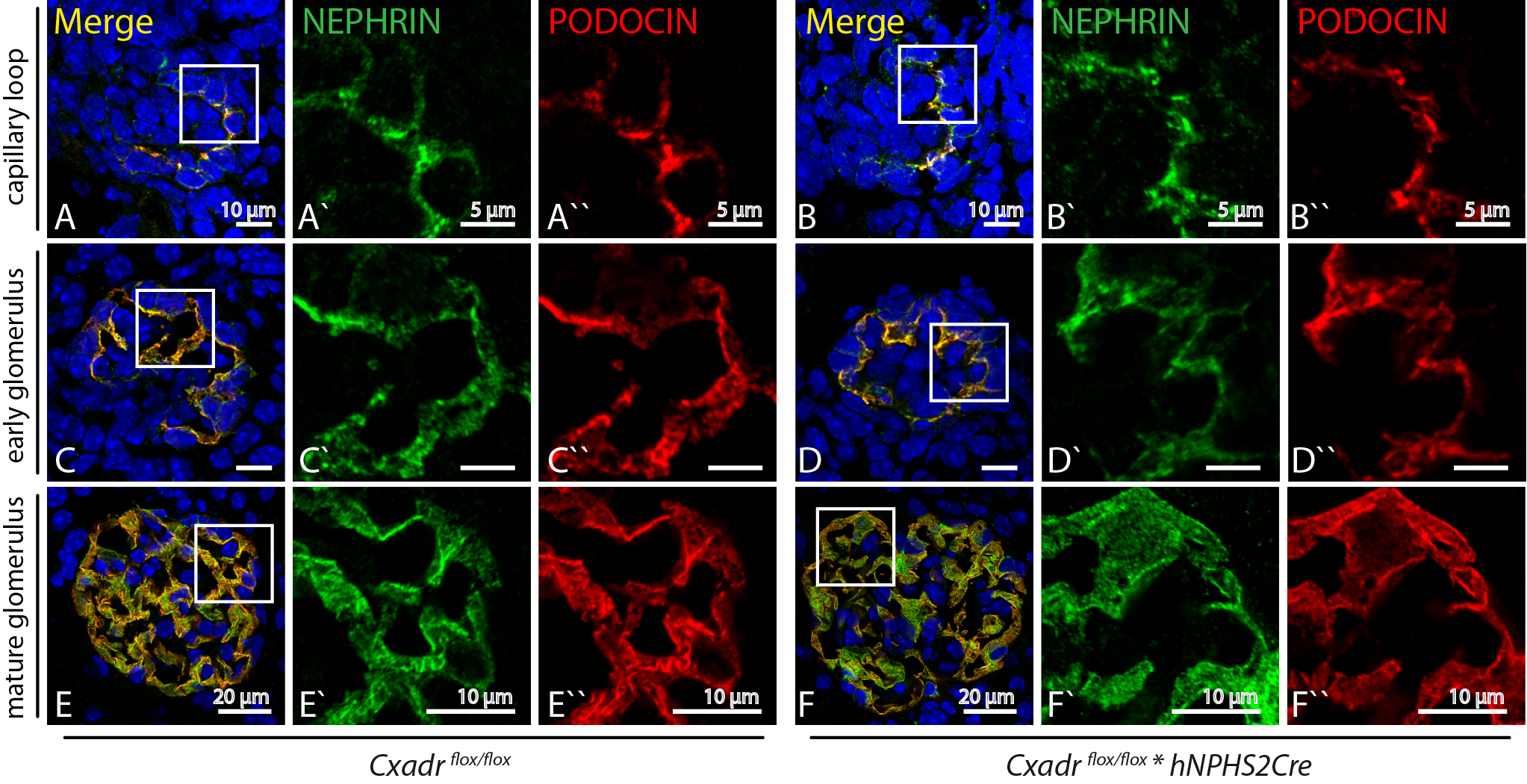

Supplement: S2 Fig — Assessment of NEPHRIN and PODOCIN expression at different developmental stages–capillary loop, early glomerulus, mature glomerulus–in control (A-A”, C-C”, E-E”) and podocyte specific Cxadr-/- animals (B-B”, D-D”, F-F”) was performed using immunofluorescence. Loss of CXADR does not lead to changes in abundance and distribution of neither NEPHRIN nor PODOCIN. (TIF) [file pone.0129424.s003.tif]

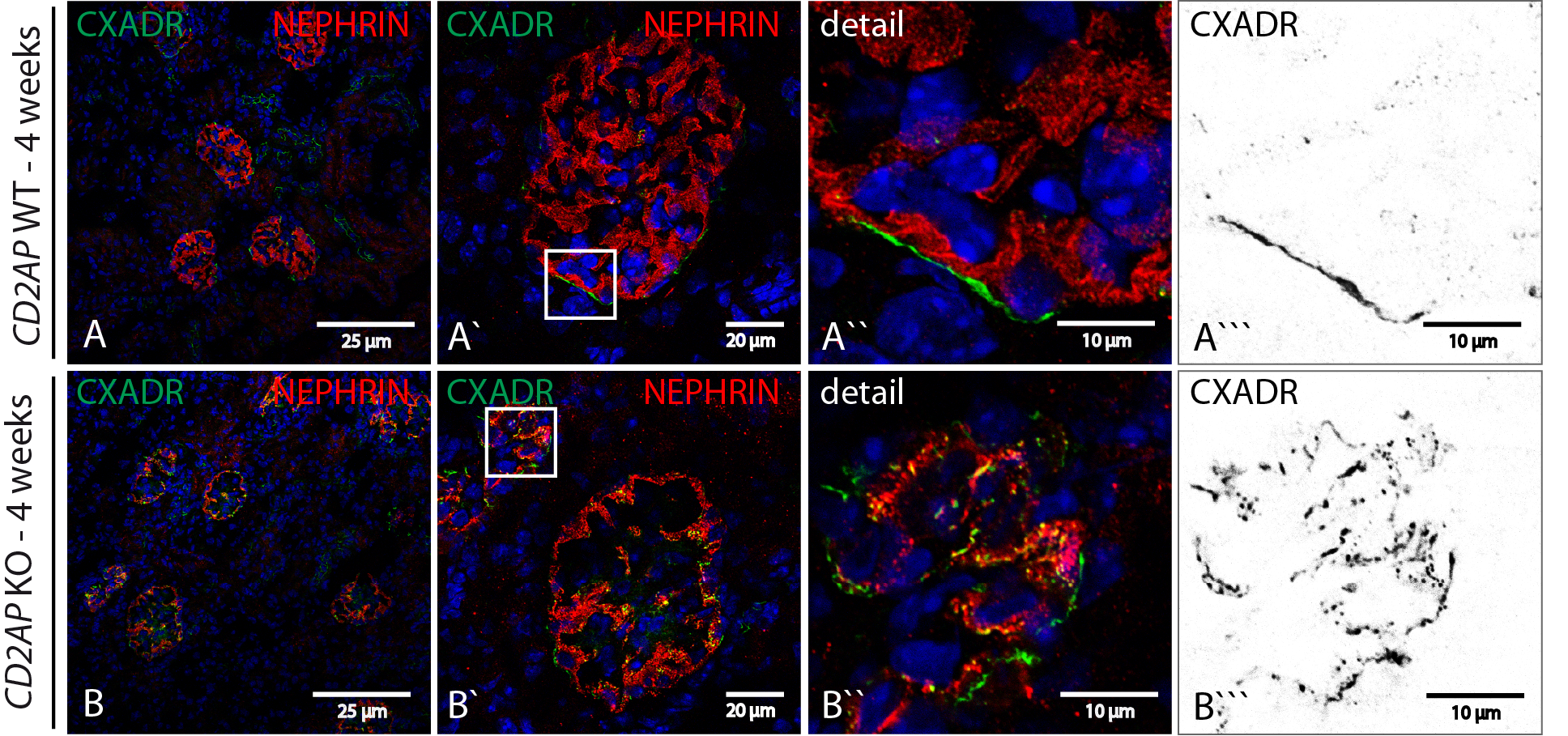

Supplement: S3 Fig — (A&B) CD2AP knockout animals exhibited a clear upregulation of CXADR expression in podocytes as demonstrated by immunofluorescence staining of CXADR and respective co-labeling with NEPHRIN. (TIF) [file pone.0129424.s004.tif]
